# Propofol Target-Controlled Infusion with the Eleveld Model in Opioid-Sparing and Opioid-Free Anaesthesia: A Narrative Review

**DOI:** 10.3390/jcm15218300

**Published:** 2026-10-25

**Authors:** Panagiotis Chatzistavridis, Anna Gkiouliava, Amalia Douma, Kassiani Theodoraki

**Affiliations:** 1 Department of Anaesthesiology, General Hospital of Athens “G. Gennimatas”, 11527 Athens, Greece; 2 Department of Anaesthesiology and Intensive Care, Aristotle University of Thessaloniki, School of Medicine, AHEPA University Hospital, 54636 Thessaloniki, Greece; 3 First Department of Anaesthesiology, School of Medicine, National and Kapodistrian University of Athens, Aretaieion University Hospital, 11528 Athens, Greece

**Keywords:** target-controlled infusion, Eleveld model, propofol, pharmacokinetics, pharmacodynamics, dexmedetomidine, ketamine, opioid-sparing anaesthesia, opioid-free anaesthesia, bispectral index

## Abstract

**Background/Objectives:** The Eleveld pharmacokinetic–pharmacodynamic (PK-PD) model is among the most extensively validated general-purpose models for propofol target-controlled infusion (TCI). It includes a binary covariate for concomitant drug administration, commonly labelled as opioid use, which reduces elimination clearance (CL) and the volume of the second peripheral compartment (V3) in an age-dependent manner. This works well for conventional opioid-based anaesthesia but is less clear for multimodal, opioid-sparing regimens built around ketamine, magnesium sulfate, dexmedetomidine and intravenous lidocaine. This review discusses how these four non-opioid adjuvants may interact with Eleveld-based propofol TCI dosing. **Methods:** We conducted a structured, non-systematic literature search of PubMed/MEDLINE, the Cochrane Library and the MDPI full-text index between May and early August 2026, combining terms for the Eleveld model and propofol TCI with terms for each adjuvant and for pharmacodynamic endpoints (EC50, Ce50, bispectral index, propofol requirement). Studies without a propofol-specific endpoint were excluded from the adjuvant-effect analysis. **Results:** Dexmedetomidine reduced the propofol concentration required for defined clinical and electroencephalographic endpoints. Magnesium sulfate reduced propofol dose requirements to a lesser extent, although concentration–response data are lacking. Ketamine’s interaction with propofol was additive rather than synergistic, and its effect on the bispectral index was dose-dependent. Lidocaine reduced propofol requirements mainly during surgical stimulation, a context-dependent effect distinct from a fixed hypnotic potency. **Conclusions:** The Eleveld model remains the best-validated general-purpose tool for propofol TCI, and its concomitant-drug covariate is a pragmatic, data-driven solution to a real gap in its source data. Clinicians should anticipate reduced propofol requirements with dexmedetomidine and, to a lesser extent, magnesium, interpret the processed-EEG response to ketamine cautiously, and expect lidocaine’s sparing effect mainly under stimulation, while titrating to clinical and electroencephalographic endpoints. Dedicated covariates for these agents, informed by prospective validation in opioid-free regimens, represent a logical next step in the model’s development.

## 1. Introduction

A patient is wheeled into the operating theatre for a laparoscopic colectomy on an enhanced-recovery pathway. The plan is dexmedetomidine and magnesium infusions starting from induction, a ketamine bolus followed by infusion, and intravenous lidocaine—no fentanyl, no remifentanil. The propofol pump is an Eleveld-based TCI device, and before the first drop is delivered, the machine asks a simple question: opioid, yes or no? Anyone who has stood at that screen with a multimodal chart in hand knows that the honest answer feels incomplete, while a more appropriate question would have to take into account many more covariates. This review grew out of that everyday moment rather than from a purely academic interest in pharmacokinetic modelling, and attempts to work out an answer.

Target-controlled infusion (TCI) lets an anaesthesiologist set a desired plasma or effect-site concentration rather than an absolute dose, with the pump translating that target into an infusion profile using an embedded population PK-PD model [1,2]. For propofol, the Marsh and Schnider models dominated clinical practice for two decades, but both were derived from comparatively narrow, largely adult, non-obese cohorts. The Eleveld model was built differently: it pooled thirty studies and more than fifteen thousand propofol concentration measurements from neonates through elderly patients, lean through morbidly obese, and added a bispectral index (BIS) sub-model so that predicted concentrations could be linked to a measurable depth-of-anaesthesia endpoint [3,4]. Prospective external validation in children, adults, older subjects and obese adults found acceptable predictive precision (median absolute performance error below 30%) in all four groups, although pharmacokinetic bias in older subjects reached −27%, exceeding the commonly used acceptance threshold of ±20% [5]. Independent studies in drug-induced sleep endoscopy [6] and in direct comparison with the Schnider model [7] have supported its use as a general-purpose tool, although a Bland–Altman analysis of predicted versus measured BIS showed wide variation between individual patients [8]. Our aim here is more specific: to consider how the model can be used effectively and safely as part of an anaesthetic strategy it was not originally built to describe, namely opioid-sparing and opioid-free anaesthesia.

Multimodal, opioid-sparing anaesthesia has not been explicitly incorporated into any TCI model. In the Eleveld model, concomitant drug administration enters as a binary covariate that reduces elimination clearance (CL) and the volume of the second, fat-free-mass-scaled peripheral compartment (V3); it does not alter the central volume (V1) or the pharmacodynamic parameters [4]. The covariate is binary because the source studies recorded concomitant drugs consistently enough to be modelled only as present or absent, rather than as graded doses [4]. Although it is commonly described, and labelled on many TCI devices, as an opioid switch [9], the covariate was originally defined more broadly as the presence or absence of concomitant anaesthetic drugs, such as opioids or local anaesthetics [4], a distinction that becomes relevant when intravenous lidocaine forms part of an opioid-free regimen (Section 5). Ketamine, magnesium and dexmedetomidine were not part of the source dataset in any systematic way, so the model has nothing to say about them directly. Pharmacology can nevertheless help the anaesthesiologist reason about propofol requirements when these agents, now an integral part of contemporary anaesthetic practice, are used.

## 2. Materials and Methods

This is a narrative rather than a systematic review, yet we followed a defined search strategy so that the reader can judge how the evidence was assembled. PubMed/MEDLINE and the Cochrane Library were searched between May and early August 2026, covering records published up to the date of the final search. We additionally searched the MDPI full-text index directly, because several recent clinical comparisons of the Eleveld and Schnider models had been published in MDPI journals; we acknowledge that searching a single publisher’s index in addition to the bibliographic databases may have introduced selection bias towards that publisher’s content. Search terms combined “Eleveld model”, “propofol target-controlled infusion” and “propofol pharmacokinetic pharmacodynamic model” with each of the four adjuvant terms in turn (“ketamine”, “magnesium sulfate”, “dexmedetomidine”, “lidocaine”), together with pharmacodynamic endpoint terms (“EC50”, “Ce50”, “bispectral index”, “propofol requirement”, “propofol-sparing”). Reference lists of retrieved articles and of recent reviews on general-purpose TCI models were hand-searched for additional studies, and further studies were identified during peer review. The literature search was performed, and records were screened by title and abstract and then in full text, by the first three authors (P.C., A.G. and A.D.), with disagreements resolved by discussion. Because this is a narrative rather than a systematic review, a formal record of screening numbers was not maintained and no PRISMA flow diagram is provided; the search is reported in line with recommendations for narrative reviews [10]. We included original clinical studies, meta-analyses and reviews, in English, that reported a quantitative pharmacodynamic or dose-requirement endpoint for propofol in relation to one of the four adjuvants, or that validated or compared the Eleveld model itself; conference abstracts, case reports and studies reporting only postoperative opioid consumption without a propofol-specific endpoint were excluded from the adjuvant-effect sections, although a small number of pharmacological reviews are cited for background. Because this is a narrative synthesis rather than a systematic review with pre-registered criteria, no formal risk-of-bias scoring or meta-analytic pooling was performed beyond citing the pooled estimates already published in the meta-analysis discussed in Section 4.2 [11]. Throughout this review, EC50 denotes the study-specific propofol concentration at which 50% of patients reach a defined clinical endpoint, typically estimated by the Dixon up-and-down method, whereas Ce50 denotes the effect-site concentration producing 50% of the maximal effect as a parameter of a pharmacodynamic model, such as the BIS sub-model of the Eleveld model.

**Figure 1:**
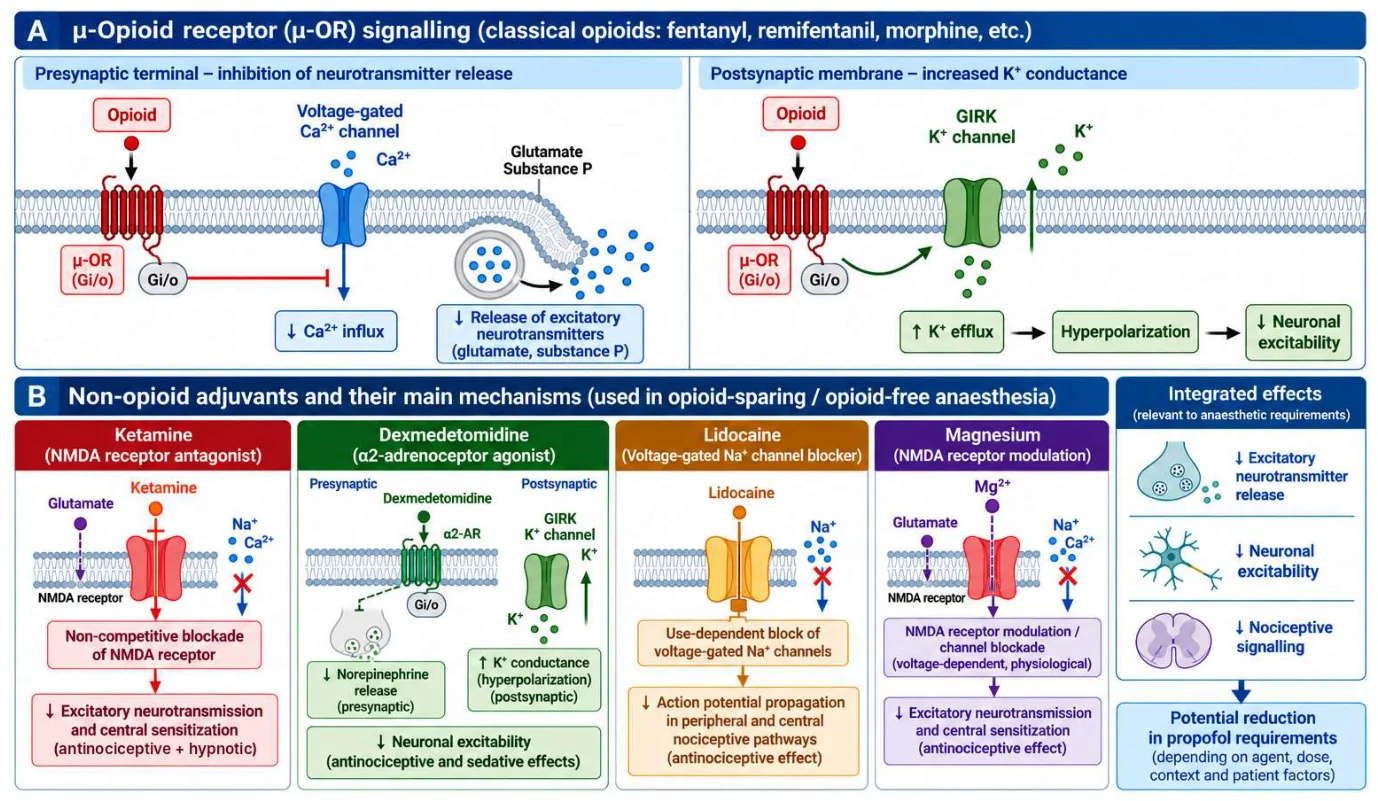
Mechanisms of action of μ-opioid receptor agonists (**A**) and of the four non-opioid adjuvants discussed in this review (**B**), and their potential relationship to propofol requirements. α2-AR, α2-adrenoceptor; GIRK, G-protein-coupled inwardly rectifying potassium channel; μ-OR, μ-opioid receptor; NMDA, N-methyl-D-aspartate. The figure was generated with ChatGPT (GPT-5; OpenAI, San Francisco, CA, USA) and reviewed and edited by the authors.

In accordance with journal policy, we disclose that generative artificial intelligence tools were used during the preparation of this manuscript. Claude (Claude Opus 5.5; Anthropic, San Francisco, CA, USA) was used to assist with literature searching, evidence synthesis and drafting of the text, and ChatGPT (GPT-5; OpenAI, San Francisco, CA, USA; accessed August 2026) was used to generate and refine Figure 1. The authors reviewed, verified and edited all AI-assisted output, including every citation against its primary source and the scientific content of Figure 1, and take full responsibility for the accuracy and integrity of the published work.

## 3. The Eleveld Model as a General-Purpose Tool

The Eleveld model was chosen as the starting point of this review because of its value as a general-purpose tool. The original 2014 pharmacokinetic model [3] and its 2018 extension with an integrated BIS pharmacodynamic sub-model [4] used allometric scaling on fat-free mass together with age-related covariates on clearance, which is what allows a single parameter set to behave sensibly across a range of body types and ages—which the Marsh and Schnider models were not designed to do [2]. Vellinga and colleagues then tested the model prospectively, enrolling 25 children, 25 adults, 25 older subjects and 25 obese adults and titrating propofol by TCI to a BIS of 40–60 [5]. Predictive precision for arterial propofol concentrations was below 30% in every group, and bias was within ±20% in children, adults and obese adults; in older subjects, however, pharmacokinetic bias reached −27%, exceeding the commonly used acceptance threshold. BIS predictions showed negligible bias but a precision of approximately 8–10 BIS units in all groups [5]. Because opioids were used in every procedure, the concomitant-opioid setting was selected for all subjects in that validation [5]. A 2026 prospective study by Driesens and colleagues, using a three-group design (adult, older, obese) for moderate-to-deep procedural sedation rather than general anaesthesia, found that the model supported completion of procedures with physiological stability in all groups; because its outcomes were procedural and physiological rather than concentration-prediction errors, it is best regarded as a clinical evaluation rather than a predictive validation [12]. Cortínez and colleagues studied obese patients, a population in which older models are known to produce miscalculations, and found acceptable performance [13]. More recent independent studies have reached similar conclusions: Linassi and colleagues compared the Schnider and Eleveld models head-to-head in a clinical cohort [7], and in a subsequent prospective study used both models to characterise how midazolam premedication shifts BIS and effect-site propofol concentration [14]. Tănase and colleagues found that the Eleveld model was not inferior to the Schnider model for propofol sedation during drug-induced sleep endoscopy [6]. Coetzee and colleagues’ 2024 Bland–Altman analysis of predicted versus measured BIS in 40 patients receiving propofol–remifentanil total intravenous anaesthesia showed a small overall bias (median prediction error −1.9 and median absolute prediction error 10 BIS units) but wide individual ranges (median prediction error −18.5 to 24.3 BIS units); the median absolute prediction error exceeded 10 BIS units in 47.8% of participants, and no participant’s limits of agreement met the criteria for interchangeability between predicted and measured BIS [8]. These findings are consistent with the BIS precision reported in the prospective validation [5]. This underlines that no PK-PD model can replace clinical titration, but does not undermine the model’s pharmacokinetic core.

The picture is not uniformly favourable, however. A 2025 PRISMA-based systematic review of the propofol TCI literature found that the Eleveld model, while generally offering the best predictive accuracy of the three widely used models, does not consistently outperform the Marsh or Schnider models in every population; for example, the Schnider model retains better bias control in older patients [15]. In a separate 2025 narrative overview, Absalom, one of the model’s developers, and Schnider likewise noted poorer predictive performance in older patients [16]. We read these findings as consistent with, rather than contradictory to, the case made in this review: one size does not fit all, in that no single population model is likely to be accurate in every patient. The realistic approach is to recognise where a model’s evidence is strong and to titrate more cautiously where it is weaker.

The Eleveld model has become the default in many modern pumps because of its larger and more diverse validation base. Its concomitant-drug covariate is a pragmatic, data-driven solution to a real limitation in the source cohorts (concomitant drug dosing was not recorded in enough detail to be modelled as a continuous covariate), and should not be regarded as a design flaw [4,9]. Its effect is also not fixed: because the covariate is an exponential function of age, it reduces CL by approximately 10% and V3 by approximately 38% at the age of 35 years, and by approximately 18% and 62%, respectively, at 70 years, with no effect in neonates [4]. Because V1 and the pharmacodynamic parameters are unchanged, selecting the covariate has little influence on the initial induction dose, but it reduces the amount of propofol delivered to fill the slowly equilibrating peripheral compartment during the redistribution phase and lowers the maintenance infusion rate once a pseudo-steady state is reached. The four adjuvants discussed below raise a related but distinct limitation, because they were essentially absent from the source data, and this is best seen as an open question for the model’s next iteration, in the same spirit as the obesity- and age-related extensions that brought the model to where it is now.

## 4. What the Four Adjuvants Actually Do to Propofol Requirements

Multimodal, opioid-sparing and opioid-free anaesthetic protocols typically layer dexmedetomidine and magnesium as a baseline infusion, add a ketamine bolus and infusion, and use intravenous lidocaine through the case. It is tempting to treat this whole bundle as pharmacologically equivalent to “giving an opioid”, since the clinical goal (a comfortable, haemodynamically stable, adequately anaesthetised patient) is the same. The underlying receptor pharmacology, however, is not interchangeable, and the evidence on propofol requirements specifically, as opposed to opioid or morphine consumption, which is a different endpoint, draws a fairly clear line between the four agents. Figure 1 summarises the mechanistic picture; the paragraphs below summarise the clinical evidence behind it.

Table 1 summarises this evidence, including the doses used in the studies discussed below. The following subsections describe the pharmacology and the individual studies in more detail.

### 4.1. Dexmedetomidine: The Clearest Case for a True Propofol-Sparing Effect

Of the four agents, dexmedetomidine has the most direct and repeatedly reproduced evidence of a hypnotic-sparing interaction with propofol, consistent with its α2-adrenoceptor-mediated sedative action [29]. The evidence, however, comes from studies that differ in route of administration, population, endpoint and background anaesthetic, and these differences limit generalisation. Using the Dixon up-and-down method, Guo and colleagues found that the EC50 of propofol for successful laryngeal mask insertion fell from 5.26 to 3.17 µg/mL, a reduction of approximately 40%, in 60 women undergoing modified radical mastectomy when dexmedetomidine 1 µg/kg was added to ropivacaine as a perineural adjuvant in a thoracic paravertebral block [17]. No opioid was given before laryngeal mask insertion in that study, but the contributions of systemic absorption and enhanced regional analgesia cannot be separated in its design. With intravenous administration, Peden and colleagues reported a reduced propofol dose requirement for loss of consciousness after dexmedetomidine premedication in patients receiving alfentanil [30]. During maintenance, Le Guen and colleagues used BIS-guided closed-loop propofol–remifentanil anaesthesia in 60 patients and found that an intravenous dexmedetomidine loading dose of 1 µg/kg followed by 0.5 µg/kg/h reduced the propofol requirement by approximately 30% at induction and by 29% during maintenance, without an increase in haemodynamic instability or delayed recovery [19]. In a randomised trial in 45 ASA III–IV patients undergoing carotid endarterectomy, Vetter and colleagues found that intravenous dexmedetomidine (0.4 µg/kg over 10 min, followed by 0.4 µg/kg/h) lowered the effect-site propofol concentration required for EEG burst suppression from a median of 6.0 to 4.0 µg/mL, a reduction of 33%, and halved norepinephrine requirements [18]. Burst suppression during carotid cross-clamping is, however, a deeper and more specialised endpoint than routine anaesthetic depth, and propofol in that study was delivered with the Schnider model against a background of remifentanil and fentanyl [18]. Overall, a patient receiving a clinically active dexmedetomidine infusion is likely to need less propofol to reach a given depth of anaesthesia, which points to an effect on hypnosis and not only on analgesia. The size of the reduction seen with one route, endpoint and background regimen, however, should not be assumed to apply directly to another.

### 4.2. Magnesium Sulfate: A Modest Reduction in Propofol Dose Requirements

Magnesium is a voltage-dependent NMDA-receptor antagonist with additional calcium-antagonist, sympatholytic and vasodilatory properties [31]. The best evidence comes from a systematic review and meta-analysis by Rodríguez-Rubio and colleagues, which included 20 randomised trials in its qualitative synthesis and 19 in its quantitative synthesis: perioperative intravenous magnesium reduced propofol dose requirements at induction (mean difference approximately −28.5 mg) and during maintenance (mean difference approximately −213.6 mg), alongside reductions in neuromuscular blocking agent and opioid consumption [11]. Two features limit the interpretation of these data. First, the meta-analysis searched the literature up to 2015 and included elective surgical patients of ASA physical status I–II, so its estimates may not apply to older or higher-risk patients [11]. Second, a reduction in total propofol dose does not by itself establish a hypnotic-sparing mechanism, and neither EC50 nor Ce50 was determined. The most relevant evidence therefore comes from trials in which propofol was titrated to a processed-EEG target; in these, magnesium reduced the propofol infusion rate required to maintain the BIS within a predefined range [20,21], although nitrous oxide formed part of the background anaesthetic in both. The effect is therefore a modest but consistent reduction in propofol requirements, whose mechanism (direct hypnotic potentiation, antinociception or both) remains unresolved.

### 4.3. Ketamine: An Additive Interaction and a Dose-Dependent Effect on the Processed EEG

Ketamine is frequently grouped with dexmedetomidine and magnesium in opioid-free protocols, but its interaction with propofol is pharmacodynamically different [32]. In a 1995 induction study in female patients, Hui and colleagues determined quantal dose–response curves for propofol and ketamine given in combination and found the interaction to be additive at both the hypnotic and anaesthetic endpoints, with no evidence of synergy [22], consistent with the broader observation that combinations of NMDA-receptor antagonists and GABAergic hypnotics tend towards additivity [33]. The effect of ketamine on the processed EEG is dose-dependent. Faraoni and colleagues found that ketamine 0.2 mg/kg given during stable propofol–remifentanil anaesthesia did not change the BIS [23]. At higher doses, the BIS may increase: Linassi and colleagues observed a biphasic, concentration-dependent BIS and spectral edge frequency response after a bolus of approximately 0.57 mg/kg during propofol–remifentanil TCI anaesthesia, with increases at lower effect-site ketamine concentrations [24]. Such changes reflect ketamine’s characteristic shift in EEG power towards higher frequencies [34], which processed indices calibrated for GABAergic hypnotics may interpret as lighter anaesthesia. Neither study included an independent measure of anaesthetic depth, so whether ketamine deepens hypnosis while the BIS rises cannot be established from them. What can be said is that, after higher-dose ketamine, a stable or rising BIS should not be taken as evidence that ketamine has had no effect, and that propofol titrated to the BIS alone may be increased in response to an index change rather than a change in hypnotic state.

### 4.4. Intravenous Lidocaine: Propofol-Sparing Mainly When There Is Something to Be Spared from

Lidocaine’s effect on propofol requirements depends on whether the patient is being surgically stimulated. Hans and colleagues, using BIS-guided TCI propofol–remifentanil anaesthesia, found that a lidocaine infusion reduced propofol requirements only during surgical stimulation; in the absence of stimulation, lidocaine changed neither the BIS nor haemodynamics, which the authors interpreted as evidence of an antinociceptive rather than a primary hypnotic mechanism [25]. Altermatt and colleagues, studying BIS-guided total intravenous anaesthesia for laparoscopic cholecystectomy in 39 analysed patients, likewise found a lower maintenance propofol requirement with a lidocaine infusion (6.00 versus 7.25 mg/kg/h), with similar end-of-infusion arterial propofol concentrations in both groups, arguing against a pharmacokinetic interaction; the authors also interpreted the sparing effect as occurring mainly during surgical stimulation [26]. The median time to extubation, however, was longer in the lidocaine group (11.0 versus 8.3 min) [26], which is relevant to enhanced-recovery pathways. In a different setting, in 90 patients undergoing upper gastrointestinal endoscopy under clinically titrated propofol sedation with fentanyl, intravenous lidocaine (1.5 mg/kg followed by 1.5 mg/kg/h) reduced total propofol consumption from approximately 131 to 98 mg, without delaying recovery [28]. Gottschalk and colleagues showed that systemic lidocaine lowers the BIS in the presence of midazolam but not in its absence, again pointing to a context-dependent, background-sensitive effect rather than a fixed hypnotic potency [27]. In practice, lidocaine’s propofol-sparing contribution should be expected mainly during surgical stimulation.

### 4.5. Safety Considerations

Each of these four agents carries its own risks, and the propofol-sparing or opioid-sparing properties discussed above should not be read as an endorsement of higher doses. The α2-agonism through which dexmedetomidine lowers propofol requirements also produces its best-known adverse effects, bradycardia and hypotension [29]. In the carotid endarterectomy trial discussed above, bradycardia occurred in three patients receiving dexmedetomidine and in one control patient, and was readily treated [18]. Magnesium sulfate can potentiate non-depolarising neuromuscular blockade and accumulates in renal impairment, so dosing should be adjusted, and serum concentrations considered, in patients with impaired renal function [31]. Ketamine’s psychotomimetic and emergence effects are dose-related and become more relevant at the upper end of the dose range than at the low, analgesia-focused doses used in most of the studies cited here [32]. Intravenous lidocaine has a narrow margin between an antinociceptive infusion and systemic local anaesthetic toxicity, particularly with cumulative dosing over a long case or in patients with hepatic impairment or cardiac conduction disease, and infusions should be dosed and monitored in line with current consensus recommendations [35]; the longer time to extubation reported with lidocaine-supplemented total intravenous anaesthesia [26] should also be considered within enhanced-recovery pathways. These risks do not alter the pharmacodynamic picture summarised in Table 1, but they mean that the decision to use these adjuvants, and at what dose, should rest on the same case-by-case risk–benefit assessment as any other part of the anaesthetic plan.

## 5. Practical Implications for Covariate Selection and Titration

The evidence reviewed above does not change what the Eleveld model was designed to predict, nor does it give reason to doubt the model in the settings in which it has been validated. It does, however, help clinicians to reason more precisely about the gap between the model’s binary concomitant-drug switch and the pharmacodynamic reality of a multimodal regimen. Three points are relevant to the choice of setting. First, the covariate was originally defined as the presence of concomitant anaesthetic drugs, such as opioids or local anaesthetics, and the model’s developers assumed their presence when simulating anaesthesia targets and their absence when simulating sedation targets [4]; when intravenous lidocaine forms part of the regimen, selecting the ‘with’ setting is therefore consistent with that definition even in the absence of an opioid, whereas for a ketamine–magnesium–dexmedetomidine regimen without lidocaine or opioids, nothing in the derivation data corresponds to the drugs being given. Second, the covariate is pharmacokinetic: it lowers CL and V3, and therefore the amount of propofol the pump delivers, but it does not modify the Ce50 of the BIS sub-model [4], whereas the effect reported for dexmedetomidine is a pharmacodynamic shift in the concentration required for a given endpoint [17,18,19]. Third, the prospective validation of the model in general anaesthesia was performed with the concomitant-opioid setting selected in all subjects [5], so the ‘without’ setting has a narrower prospective evidence base in this context. The developers also cautioned that, because only the presence or absence of these drugs was recorded, the model may not perform ideally when opioid exposure differs substantially from that in the source studies [4]. Neither setting has been validated in opioid-free multimodal anaesthesia.

In practice, this argues for a modest and transparent approach. The covariate should be set according to its original definition, that is, ‘with’ when an opioid or intravenous lidocaine is part of the regimen, as for the patient described in the Introduction. The clinician should be aware that, at the age of 35 years, this lowers modelled CL by approximately 10% and V3 by approximately 38%, and more in older patients, and therefore reduces delivered propofol mainly during the redistribution and maintenance phases rather than at induction [4]. Whichever setting is chosen, the target concentration should start towards the lower end of the usual range once dexmedetomidine, and to a lesser extent magnesium, are running at clinically active doses, and processed-EEG indices and clinical signs should guide the remainder of the titration. Ketamine deserves a specific caveat: because its effect on the BIS is dose-dependent and can dissociate from the clinical state [23,24], a stable or rising BIS after a higher-dose ketamine bolus should not prompt an automatic increase in the propofol target, and inspection of the raw EEG and density spectral array may be more informative than the index alone [34]. Lidocaine’s contribution is best anticipated once surgical stimulation begins rather than built into the starting target.

These considerations should also be tailored to the type of surgery. The Eleveld model includes covariates for age, weight, height, sex and concomitant drugs, but none for the surgical procedure, whereas the procedure can change the physiological determinants of propofol disposition and effect. Cardiac output is a determinant of early propofol concentrations [36], so procedures associated with marked haemodynamic change (thoracic surgery with one-lung ventilation, major abdominal surgery with pneumoperitoneum and positional changes, or surgery with significant blood loss) may produce concentrations that deviate from model predictions. In children, the maturation of clearance and the relationship between body size and drug disposition differ from those in adults [37], and in neuroanaesthesia, endpoints such as burst suppression or compatibility with neurophysiological monitoring may take precedence over a standard BIS target [18]. A multimodal regimen should therefore be integrated with procedure-specific physiological goals, so that organ function and the patient’s individual physiology are respected, rather than applying a single target strategy across thoracic, abdominal and paediatric surgery [2].

## 6. Limitations and a Direction for Future Model Development

This is a narrative rather than a systematic review, and the search strategy in Section 2, while structured, is not a substitute for a pre-registered systematic review with formal risk-of-bias assessment; the quality of evidence behind each adjuvant also varies—dexmedetomidine rests on concentration–response and processed-EEG-guided data obtained in heterogeneous settings, magnesium on dose-requirement data from a meta-analysis without concentration–response measurements, ketamine on interaction and processed-EEG studies, and lidocaine on dose-requirement data that depend on surgical stimulation. Because screening numbers were not formally recorded, study selection may also be subject to selection bias. We are not aware of any published study that has directly measured Eleveld-model predictive performance in patients receiving a full multimodal, opioid-free regimen of the kind described here; that is the most immediately useful study someone could run, and it would sit naturally alongside the obesity- and paediatric-focused validation work that has already extended the model’s reach.

We have identified two noteworthy gaps in the literature. First, much of the pharmacodynamic evidence cited above was generated against a background that itself included an opioid, a local anaesthetic or both: Faraoni and colleagues’ ketamine data and Hans and colleagues’ lidocaine data were obtained during propofol–remifentanil anaesthesia, and Altermatt and colleagues’ lidocaine data with fentanyl [23,25,26]; the intravenous dexmedetomidine data of Le Guen and colleagues and of Vetter and colleagues were obtained with remifentanil, and in the latter also fentanyl [18,19]; and the largest dexmedetomidine EC50 reduction was observed with a ropivacaine paravertebral block [17]. None of these studies used the Eleveld model to deliver propofol. The effect sizes we cite were therefore not obtained in a genuinely opioid-free setting, and their magnitude in a fully opioid-free multimodal regimen remains an extrapolation rather than a direct measurement. This applies particularly to lidocaine, which may itself fall within the model’s concomitant-drug covariate [4], so that part of its effect could already be represented in the ‘with’ setting. Secondly, and more fundamentally, none of the studies reviewed here tested dexmedetomidine, magnesium, ketamine and lidocaine administered together with propofol; each agent’s contribution has been isolated only one at a time. Whether the combined pharmacodynamic effect of a full multimodal regimen on propofol requirements is additive, supra-additive or sub-additive is not established and would require a dedicated interaction study, ideally analysed with response-surface methods [33,38], rather than an assumption built from single-agent data.

Such a study could follow the design already used to validate the model in obese and older patients [5,13]: a prospective cohort of patients receiving a standardised multimodal, opioid-free regimen (for example, dexmedetomidine and magnesium infusions with a ketamine bolus and infusion), with arterial propofol sampling and continuous BIS or other processed-EEG recording, compared against a conventional opioid-based cohort matched for age, weight and surgical procedure. The primary endpoint would be the same one used in the original validation studies—predictive precision (median performance error and median absolute performance error) for measured versus Eleveld-predicted plasma or effect-site concentration and BIS—with dexmedetomidine and magnesium exposure analysed as candidate covariates on CL, V3 and the Ce50 of the BIS sub-model, in the same way that concomitant drug administration was originally analysed [4]. A sample size in the range of the 25-patients-per-group design used in the original prospective validation [5] would likely be sufficient to detect a covariate effect of the magnitude reported for dexmedetomidine’s effect [17,18,19], though a larger cohort would be needed to resolve the more modest magnesium effect [11]. A future model refinement, following the same logic that let concomitant drug administration into the model in the first place, might then treat dexmedetomidine or magnesium exposure as an additional covariate rather than trying to force multimodal regimens through the existing concomitant-drug switch. This is no longer a purely hypothetical extension: general-purpose pharmacokinetic–pharmacodynamic models have now been developed for remifentanil and, notably, for dexmedetomidine itself, alongside the Eleveld propofol model [9,16], which raises the more ambitious possibility of a formally linked multi-drug interaction model rather than a single additional covariate added to the existing one. At the same time, Absalom and Schnider’s own 2025 assessment of the field is that further proliferation of new pharmacokinetic models is unlikely, on its own, to be clinically beneficial, and that effort is better spent on clinician education and individualised titration [16], a conclusion we find broadly compatible with the practical, monitoring-led approach recommended in Section 5. Until dedicated multimodal validation or interaction data exist, the approach outlined there is offered as a reasonable bridge, grounded in the pharmacodynamic literature that does exist, rather than as a substitute for it.

## 7. Conclusions

The Eleveld model remains, in our view, the best available general-purpose pharmacokinetic–pharmacodynamic tool for propofol TCI, with a validation record now spanning children, adults, older patients and the obese that no earlier model can match, albeit with greater pharmacokinetic bias in older patients. Its binary concomitant-drug covariate is a sound solution given the data it was built from, not a defect. The rise in multimodal, opioid-sparing anaesthesia simply asks a question the original dataset could not answer on its own: what happens pharmacodynamically when ketamine, magnesium, dexmedetomidine and lidocaine replace the opioids used in most of the source studies? The best answer the current literature allows us to give is that dexmedetomidine reduces the propofol concentration required to reach defined endpoints and magnesium modestly reduces propofol dose requirements, and both effects should be anticipated, while ketamine interacts additively with propofol and can confound processed-EEG monitoring, and lidocaine acts mainly through antinociception during surgical stimulation. This is not an argument against the Eleveld model. Rather, it is a case for extending the same careful, data-driven approach that built it in the first place to the anaesthetic practice of today.

## Figures and Tables

**Table 1:** Summary of the four non-opioid adjuvants discussed in this review: primary mechanism; route, population, endpoint and sample size of the key studies, with their effect on propofol requirements; representative dosing; and strength of the supporting evidence. BIS = bispectral index; Ce = effect-site concentration; EC50 = concentration effective in 50% of patients; LMA = laryngeal mask airway; RCT = randomised controlled trial; ↓ = reduction; → = change from the control to the intervention group.

Adjuvant	Primary Mechanism	Key Studies (Route, Population, Endpoint, *n*) and Effect on Propofol Requirement	Typical Dose in the Studies Cited	Evidence Strength	Ref.
Dexmedetomidine	α2-adrenoceptor agonism (presynaptic + postsynaptic)	Le Guen: IV, BIS-guided closed-loop propofol–remifentanil, adult surgical patients (*n* = 60): maintenance dose ↓29%. Vetter: IV, EEG-titrated burst suppression in carotid endarterectomy, ASA III–IV (*n* = 45): Ce ↓33% (6.0 → 4.0 µg/mL). Guo: perineural (paravertebral block with ropivacaine), EC50 for LMA insertion, women undergoing mastectomy (*n* = 60): ↓~40% (5.26 → 3.17 µg/mL)	IV: 0.4–1 µg/kg loading + 0.4–0.5 µg/kg/h infusion; perineural: 1 µg/kg with ropivacaine	Moderate—RCTs with heterogeneous routes, endpoints and background agents	[17,18,19]
Magnesium sulfate	Voltage-dependent NMDA-receptor channel block	Meta-analysis of RCTs (20 trials in qualitative and 19 in quantitative synthesis; ASA I–II elective surgery; search to 2015): ↓ induction (≈−28.5 mg) and maintenance (≈−213.6 mg) dose. BIS-guided RCTs: ↓ infusion rate at constant BIS. EC50/Ce50 not determined	Variable across trials (commonly a bolus ± continuous infusion)	Moderate for dose reduction; no concentration–response data	[11,20,21]
Ketamine	Non-competitive NMDA-receptor antagonism	Hui (induction, female patients): additive, not synergistic, at the hypnotic endpoint. During stable propofol anaesthesia: no BIS change after 0.2 mg/kg (Faraoni); BIS increase after ≈0.57 mg/kg (Linassi)	0.2–0.6 mg/kg bolus ± infusion	Established additive (not synergistic) interaction; dose-dependent processed-EEG effect	[22,23,24]
Lidocaine (i.v.)	Voltage-gated Na^+^ channel blockade	Hans (BIS-guided TCI propofol–remifentanil): ↓ only during surgical stimulation. Altermatt (BIS-guided TIVA, *n* = 39): ↓ maintenance dose (6.00 vs. 7.25 mg/kg/h); longer time to extubation. Arun (endoscopic sedation, *n* = 90): ↓ total dose (≈131 → 98 mg). Gottschalk: BIS ↓ only in the presence of midazolam	1.5 mg/kg bolus + 1–2 mg/kg/h infusion	Moderate—several RCTs	[25,26,27,28]

## Data Availability

No new data were created or analysed in this review. Data sharing is not applicable to this article.
